# The Effect of Polysorbate 20 on Solubility and Stability of Candesartan Cilexetil in Dissolution Media

**DOI:** 10.1208/s12249-014-0109-8

**Published:** 2014-05-29

**Authors:** Katarzyna Hoppe, Małgorzata Sznitowska

**Affiliations:** Department of Pharmaceutical Technology, Medical University of Gdansk, Hallera 107, Gdansk, 80-416 Poland

**Keywords:** candesartan, dissolution, polysorbate, solubility, stability

## Abstract

The addition of polysorbate 20 (T20) is required to achieve “sink” conditions during a dissolution test for tablets with candesartan cilexetil (CC). Polysorbate 20 (0.35%–0.7% w/w) added to 0.05 mol/L of phosphate buffer pH 6.5 dramatically increased the apparent solubility of the drug from 0.8 μg/ml even to 353 μg/ml, while its effect in lower pH or in water was much smaller (20 μg/ml in pH 4.5). The increased concentration of phosphate salts (0.2 mol/l) at pH 6.5 in the presence of 0.7% of polysorbate 20, resulted in further increase of candesartan cilexetil solubility to 620 μg/ml. The change of pH from 1.2 to 7.4 resulted in a 1.5-fold increase of the activation energy and, depending on temperature, 8–14-fold decrease of the degradation rate. When polysorbate 20 increased the activation energy 2-fold, independent of pH, it protected candesartan cilexetil from degradation; however, this effect was temperature dependent and was very small at 310 K—the degradation rate in pH 6.5 decreased by 13% only. It was calculated that in the phosphate buffer pH 6.5 with polysorbate, one can expect during 24 h the degradation at the level of 9.3%, thus a flow-through dissolution apparatus was recommended for testing prolonged release dosage forms.

## INTRODUCTION

Dissolution testing has been employed as a quality control procedure in pharmaceutical production, and the objectives of dissolution testing vary during the life cycle of a dosage form. In product development, it is used to assist in the selection of a candidate formulation, in research to detect the influence of critical manufacturing variables, such as the binder effect, mixing effect, granulation procedure, coating parameters, and excipient type [1]. It, therefore, becomes apparent that the dissolution data derived from the physico-chemically and hydro-dynamically defined conditions is required in order to compare various *in vitro* dissolution data and to be able to use such results as a surrogate for possible *in vivo* bioequivalence testing and *in vitro*-*in vivo* correlations (IVIVC) [1, 2].

Dissolution characteristics of oral formulations should be evaluated using the test media within the physiological pH range of 1.2–6.8 (1.2–7.5 for modified-release formulations). Selection of the dissolution medium is based, in part, on the solubility data and its stability measured at the pre-formulation stage [2]. In order to ensure that the dissolution test is measuring the properties of drug release without the limitations imposed by the experimental conditions, the medium is selected to ensure that the concentration gradient remains large and “sink” conditions occur for the duration of the test. “Sink” conditions are achieved when the drug solubility is 10 times the total concentration of the drug in a vessel or at least greater than three times [2, 3].

**Fig. 1:**
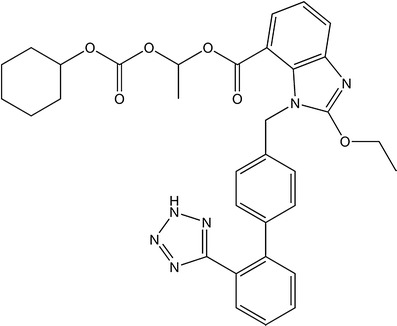
Chemical structure of candesartan cilexetil (1*RS*)-1-[[(Cyclohexyloxy)carbonyl]oxy]ethyl 2-ethoxy-1-[[2′-(1*H*-tetrazol-5-yl)biphenyl-4-yl]methyl]-1*H*-benzimidazole-7-carboxylate

Candesartan cilexetil (CC), whose chemical structure is presented in Fig. 1, is a potent and long-acting angiotensin II receptor blocker, commonly used to treat hypertension in monotherapy or in combination with other antihypertensive agents, such as diuretics. CC is available as nonmodified release tablets in doses of 8, 16, or 32 mg (an original product from Astra Zeneca and generics from *e.g*., Sandoz, Krka, Zentiva). CC is a pro-drug, which is rapidly and completely converted into an active moiety, candesartan, during a gastrointestinal absorption [4]. Miyabashi [5] has identified candesartan and desethyl candesartan, as a metabolite of CC, in plasma samples.

CC is practically insoluble in water (<0.05 μg/mL) and is a highly lipophilic compound. The partition coefficient (*C*_octanol_/*C*_water_) at pHs 1.1, 6.9, and 8.9 is >1,000, indicating high hydrophobicity [4, 6]. Solubility of CC in a simulated gastric fluid and intestinal fluid has been reported as 0.6 and 8.6 μg/mL, respectively [7]. Low solubility of CC across the physiological pH range resulted in an incomplete absorption from the gastrointestinal tract and, hence, it is reported to have an oral bioavailability of about 15% [4, 8]. Based on CC’s solubility across the physiological pH and absorption characteristics, it is classified in the Biopharmaceutics Classification System as a class II drug [8].

Solubility of CC in various surfactant solutions and oils has been reported. It is slightly soluble in Miglyol 812 or Labrafac and sparingly soluble in Labrafil. Polysorbate 80, macrogol 400, Cremophor, Labrasol, and Transcutol P were used to solubilize CC in water in order to develop a self-microemulsifying formulation as a new dosage form [8]. In a phosphate buffer (PBS) pH 6.5 containing 0.7% of polysorbate 20 (T20) and in PBS pH 6.8 with 1% of sodium lauryl sulfate, the solubilities have been reported as 125.0 μg/mL [9] and 1.0 μg/ml [10], respectively. This indicates how important a choice of additives in dissolution media is when “sink” conditions have to be achieved.

In its solid state, CC is stable against temperature, moisture, and light but undergoes degradation when subjected to acidic, basic, aqueous hydrolysis, and oxidative conditions [4, 11]. Rao *et al.* [12] have determined and identified several impurities of CC in alkaline or acidic conditions and under oxidative and hydrolytic stress. CC is highly sensitive to bases and is degraded to candesartan and transesterification products (methylcandesartan, ethylcandesartan, hydroxyethylcandesartan) [11, 12]. Mohan *et al.* [13] determined and identified five degradation products of CC in tablets. Subjected to 40°C and 75% RH, the parent drug molecule underwent reactions which resulted in the formation of desethylcandesartan cilexetil and ethylcandesartan cilexetil as main impurities.

Poor solubility and stability of the drug require special conditions when dosage forms are subjected to dissolution tests and dissolution medium should be carefully chosen. From the collected data, a summary of the CC dissolution media recommended in literature was made (Table I). For the purpose of the *in vitro* release study from CC dosage forms (tablets, nanoemulsions, nanosuspensions, polymeric micelles, self-microemulsifying drug delivery systems (SMEDDS)), dissolution media in the pH range from 1.0 to 7.2 were selected. Considering the solubility and stability of CC, a dissolution media such as the phosphate buffers pH 7.2, pH 6.8 (with 0.1% T20), and pH 6.5 (with 0.02% T20) were recommended. Despite the indicated poor solubility [6] and susceptibility to hydrolysis into desethylcandesartan [11] in the acidic medium, dissolution tests were carried out in the acetate buffer pH 4.5, 0.1 mol/L HCl and simulated gastric fluid at pH 1.2.

**Table I:** Dissolution Media Used for Biopharmaceutical *In Vitro* Studies of Candesartan Cilexetil (CC) in Dosage Forms

Dosage form of CC	Dissolution medium		References
	Recommended	Other investigated solutions	
Tablets	0.35/0.7% T20 in 0.05 mol/L PBS pH 6.5	–	[14]
Tablets	PBS pH 7.2	PBS pH 6.5	[19]
		Water	
		0.1 mol/L HCl	
Tablets	1% SLS pH 6.8	0.1 mol/L HCl	[20]
		PBS pH 7.0	
Fast-dissolving tablets	Sorenson’s buffer pH 6.8	–	[21]
Fast-dissolving tablets	0.1% T20 in PBS pH 6.8	–	[22]
Nanosuspension/tablets	PBS pH 6.8	0.1 mol/L HCl	[10]
		Acetate buffer pH 4.5	
		Water	
Nanosuspension	0.7% T20 in 0.05 mol/L PBS pH 6.5	–	[9]
Nanoemulsion	0.1 mol/L HCl	–	[23]
	PBS pH 6.8		
	Artificial intestine juice (AIJ) pH 6.8		
Microspheres	PBS pH 6.8	–	[24]
Polymeric micelles	Simulated gastric fluid (SGF) pH 1.2, after 2 h pH 7.2	–	[7]
Solid lipid nanoparticles	0.1 mol/L HCl	–	[25]
	PBS pH 6.8		
SMEDDS	0.02% T20 in 0.05mol/L PBS pH 6.5	–	[8, 26]

*CC* candesartan cilexetil, *SLS* sodium lauryl sulfate, *PBS* phosphate buffer solution, *T20* polysorbate 20, *SMEDDS* self-microemulsifying drug delivery system, *HCL* hydrochloric acid

The aims of the present study were to investigate the solubility of CC in the selected dissolution media and to indicate the kinetics of CC degradation in the aqueous solution as a function of pH, temperature, surfactant concentration, and buffer type at different pHs (1.2–7.4). The obtained data should be helpful in selecting an appropriate acceptor fluid for dissolution testing.

## MATERIALS AND METHODS

### Chemicals

Candesartan cilexetil (CC) was kindly donated by Polpharma S.A. Pharmaceutical Works (Starogard, Poland). Acetonitrile (Merck, Darmstadt, Germany) and methanol (POCh, Gliwice, Poland) were of HPLC grade. Sodium hydroxide, dipotassium phosphate, and phosphoric acid were purchased from POCh as reagents for preparation of buffered solutions. High-purity water was prepared by use of a Millipore Elix 3 (Millipore, Bedford, MA, USA) water purification system. Phosphate-buffered saline (PBS 0.05 mol/L) pH 4.5, pH 6.5, and pH 7.4 were prepared by dissolving potassium dihydrogen phosphate in water and adjusting it to a desired pH with 0.1 mol/L of sodium hydroxide. In the same way, PBS pH 6.5 at concentrations of 0.01 and 0.2 mol/L was prepared. The pH was measured at room temperature (pH-meter (type 350), Orion Research, Boston, USA). Ionic strengths were 0.05, 0.079, and 0.123 mol/L for pH 4.5, pH 6.5, and pH 7.4 buffer solutions (0.05 mol/L), respectively. Hydrochloric acid (HCl; POCh, Gliwice, Poland) was used in the concentration of 0.1 mol/L (the measured pH was 1.2). Polysorbate 20 (T20, Tween 20) was purchased from Sigma Aldrich (Steinheim, Germany). Solutions containing either 0.35% or 0.7% (w/w) of T20 were prepared by dissolving the surfactant in 0.1 mol/L HCl or 0.05 mol/L PBS pHs 4.5 and 6.5.

### HPLC Analysis

Chromatographic separation and quantitative analysis were performed using HPLC apparatus (Merck-Hitachi, Darmstadt, Germany) equipped with C18 column (5 μm LiChrosphere 250 × 4 mm, Merck) and UV–Vis detector at 215 nm. A mixture of 0.05 mol/L of phosphate buffer pH 3.0:acetonitrile:methanol (30:50:20 v/v) was used as a mobile phase. Prior to injection, the samples were diluted with methanol.

### HPLC Validation

The method was validated for the limit of quantification (LOQ) and limit of detection (LOD), linearity, accuracy, and precision. A stock solution of CC (1 mg/ml) was prepared in methanol, and further dilution was also made in the same solvent.

#### Linearity

The linearity of the method was checked by analyzing six solutions of CC in the concentration range of 0.2–10.0 μg/mL and prepared in triplicate. The least-squares linear regression analysis of the peak area and concentration data was performed.

#### Limits of detection (LOD) and quantification (LOQ)

The LOQ and LOD of the method were determined, based on signal-to-noise ratios. LOD was determined as the lowest CC concentration which can be detected by the HPLC system producing a signal-to-noise ratio of about 3. LOQ was the concentration producing a signal-to-noise ratio of 10.

#### Accuracy and precision

The intra-day and inter-day accuracy and precision were established using samples at the concentrations of 10.0, 5.0, and 1.0 μg/mL in triplicate. The accuracy was defined as the recovery percentage, and precision was expressed as the percent relative standard deviation (RSD).

### Solubility Determination

CC was added, in excess, into pure solvents (water, 0.1 mol/L HCl, PBS 4.5, PBS 6.5) or to the solvents with addition of 0.35% or 0.7% (w/w) of T20 and the resulting suspensions were shaken for 24 h under thermostated circumstances (310 K, *i.e.*, 37°C) to obtain the solubility equilibrium (no further increase in dissolved drug concentration was observed after 48 h). The amount of T20 was added according to FDA dissolution methods [14]. To demonstrate the effect of PBS concentration with pH 6.5 on CC solubility, the experiment was performed in 0.01, 0.05, and 0.2 mol/L PBS (with and without T20). The samples of saturated solutions were collected after filtration through a 0.22 μm filter (cellulose acetate filter, Sartorius, Goettingen, Germany) and diluted appropriately with methanol to prevent the crystallization of the solute. The concentrations of CC were analyzed by the HPLC method.

### Stability Study

Degradation of CC was studied in solutions prepared with 0.1 mol/L HCl (pH 1.2), 0.05 mol/L PBS (pHs 4.5, 6.5, and 7.4), and in water (W-6.8) (pH 6.8; measured in 10 μg/ml aqueous solution of CC). Experiments were repeated in solutions containing 0.35% or 0.7% of T20 in 0.1 mol/L HCl and PBS pH 4.5 or 6.5. The solutions were prepared at the concentration of 10 μg/mL by diluting a 1 mg/mL methanolic stock solution; the final concentration of methanol in the solutions was approximately 1%. Solutions of CC in methanol were also subjected to stability studies.

The investigated solutions were stored for 14 days in temperature-controlled areas at 277, 298, and 310 K (4°C, 25°C, and 37°C). Aliquots were withdrawn at appropriate time intervals and, after diluting with methanol, were immediately analyzed for the remaining CC. The observed pseudo-first-order degradation rate constants, *k*_obs_, were calculated from the slopes of the natural-logarithmic plots of the remaining drug fraction *versus* time in accordance with Eq. (1) [15]: where *C*_0_ is the initial concentration and *C*_*t*_ is the concentration of CC at time *t*:

1 $$ \ln \left(\frac{C_t}{C_o}\right)=-{k}_{obs}t $$

Coefficient of determination, *r* ^2^ was used in linear regression analysis to evaluate how the experimental data fit the pseudo-first-order degradation kinetics.

The number of *C*_*t*_ measurements taken over the time span from *t*_0_→*t* ranged at 5. The Arrhenius factor (*A*) and the activation energy (*E*_a_) for CC degradation were determined from a plot of ln (*k*_obs_) = *f* (1/T) according to Eq. (2), using the least-squares regression: where *R* is the universal gas constant and *T* is the absolute temperature (K).

2 $$ \ln {k}_{obs}= \ln A-\frac{E_{\mathrm{a}}}{ RT} $$

## RESULTS AND DISCUSSION

### Validation of HPLC

**Fig. 2:**
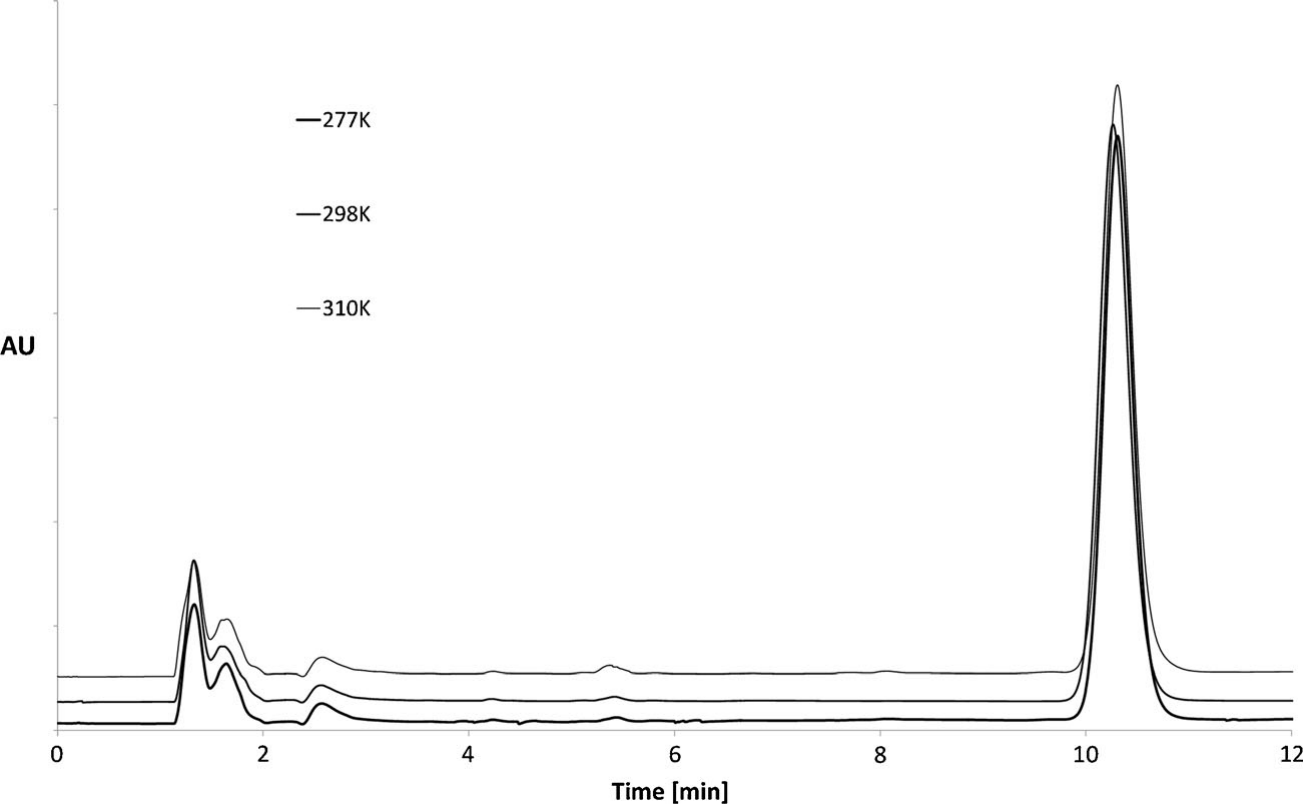
The HPLC chromatograms of candesartan cilexetyl methanolic solutions after 14 days storage at temperature 277, 298, and 310 K

Concentrations of CC in solutions were measured using the HPLC method previously described by Lunn [16] and modified for the purpose of this study. An example of the chromatogram is presented in Fig. 2. The least-squares linear regression analysis revealed the linearity in the concentration range of 0.2–10.0 μg/mL: the regression line equation was *y* = 58.076*x* + 5.99 and the coefficient of determination *r* ^2^ was 0.9994. The LOD was 0.05 μg/mL and the LOQ 0.17 μg/mL. The method was characterized by the acceptable precision (≤6.0% RSD) and accuracy (94%–104%).

### Solubility

Solubilities of CC in the aqueous solvents (HCl, PBS 4.5, PBS 6.5, and W-6.8), also with an addition of 0.35% or 0.7% of T20 at temperature 310 K are shown in Table II (the relative standard deviations (RSD) between the repeated determinations were not larger than 10%). According to the pharmacopoeial classification, CC is practically insoluble in water since its solubility was below 100 μg/ml [4]. The pH-dependent solubility profile of CC compound was observed, however, with only slightly better solubility at higher pH (0.23 μg/ml at pH 1.2 and 1.40 μg/ml at pH 6.8).

**Table II:** The Effect of Polysorbate 20 (T20) on Solubility of Candesartan Cilexetil (Μg/ml) in Dissolution Media at Temperature 310 K (*n* = 3; mean ± RSD)

Solvent	pH value	T20 (% w/w)		
		0	0.35	0.7
HCl	1.0	0.23 (±47.8)	6.2 (±15.3)^b^	11.5 (±9.5)^c^
0.1 mol/L				
PBS 4.5	4.5	0.51 (±37.2)^a^	7.8 (±12.1)^b^	20.1 (±8.1)^c, d^
0.05 mol/L				
PBS 6.5	6.5			
0.01 mol/L		<0.05	168.8 (±2.3)	313.7 (±7.8)^c, d^
0.05 mol/L		0.8 (±25.8)^a^	192.9 (±3.9)^b^	353.4 (±9.4)^c, d^
0.2 mol/L		1.3 (32.1)^a^	201.5 (±5.0)^b^	620.3 (±6.5)^d^
Water	6.8	1.4 (±27.2)^a^	7.4 (±7.3)^b^	10.3 (±6.9)^c^

Statistically significant difference (*p* < 0.05)

*HCL* hydrochloric acid, *PBS* phosphate buffer solution, *T20* polysorbate 20

^*a*^Compared with HCl

^*b*^Compared with PBS 6.5 (0.01 mol/L)

^*c*^Compared with PBS 6.5 (0.2 mol/L)

^*d*^Compared with HCl

As determined by Satturwar *et al.* [7], the solubilities of CC at temperature of 310 K in a simulated gastric fluid with pH 1.2 (0.6 μg/ml) and in a simulated intestinal fluid with pH 7.2 (8.6 μg/ml), although larger than reported in the present study, are consistent with respect to pH-dependent solubility. This relationship can be supported by an acid–base equilibrium reported by Cagigal *et al.* [17], who determined p*K*_*a*_ at the value 6.3 for CC and attributed it to the deprotonation of the tetrazole group classifying CC as a weak acid. The pH-dependent solubility of CC was studied also by Nekkanti *et al.* [10], who concluded that the solubility decreases with an increasing pH, however, that was not demonstrated by the experimental results since, for micronized CC, no effect of pH was observed and, for nonmicronized substance, the solubility at pHs 4.5 and 6.8 was the same.

The solubility enhancement of poorly soluble drugs, required for the dissolution studies from drug formulation, can be achieved by using surfactants. In the present work, the solubility of CC in water or buffers was enhanced using T20 according to FDA recommendation (Table I). The surfactant was used in concentrations much above the critical micellar concentration (CMC for T20 is, according to different literature sources, 0.001%–0.007% *w*/*v*). T20 has significantly increased the solubility of CC in all solvents, with a larger effect (1.3–2.5 times larger) observed at higher concentration, *i.e.*, 0.7%. Since the drug is solubilized in micelles, the solubility should be considered as an apparent solubility.

At least 2.5 times lower solubility of CC (125 ± 6.9 μg/ml) in PBS pH 6.5 containing 0.7% of T20, than in the present study, was reported by Detroja *et al.* [9], but the experiment was performed at room temperature. Thus, the results obtained in the present study at 310 K are coherent.

Although solubilities of CC in PBS at pH 4.5 or 6.5 without a surfactant were comparable, but upon addition of T20, a dramatic increase (200–500-fold) of CC solubility was observed at pH 6.5, while the effect was much smaller at pH 4.5 (15–40-fold). The effect of T20 on CC solubility in water (pH 6.8) was also not so spectacular. It is interesting to note that another surfactant, namely sodium lauryl sulfate, added to PBS with pH 6.8 in the concentration of 1% did not exhibit such large solubilization effect [10].

The results suggest that micellar solubilization is more effective in the presence of phosphate ions at higher pH. To elucidate this phenomenon, the relationship between PBS concentration (0.01–0.2 mol/L) and solubility of CC in the presence of T20 was studied. It was observed (Table II) that an increased ionic strength of PBS allows to increase drug solubility, especially at higher concentration of T20. This effect is not so large, however, in 0.35% of T20 solution and does not explain a huge difference obtained when T20 is added either to PBS or water. Since T20 is a nonionic surfactant, one cannot explain, without conducting further investigation, the reason for such large solubilization effect only in PBS pH 6.5 but not in water or in PBS pH 4.5.

Results demonstrate that at least 125 and 500 ml, respectively, of 0.05 mol/L of PBS at pH 6.5 with 0.35% of T20 is required for 8 and 32 mg tablets to achieve a minimum “sink” conditions (approximately 30% of the saturation concentration) in dissolution studies. If T20 is used in concentration 0.7%, the volumes could be further reduced. On the other hand, the “sink” conditions for 8 mg tablets tested in HCl or PBS 4.5 would require about 1–2 L of the medium containing 0.7% of T20.

### Stability Study

#### Methanolic solutions

**Fig. 3:**
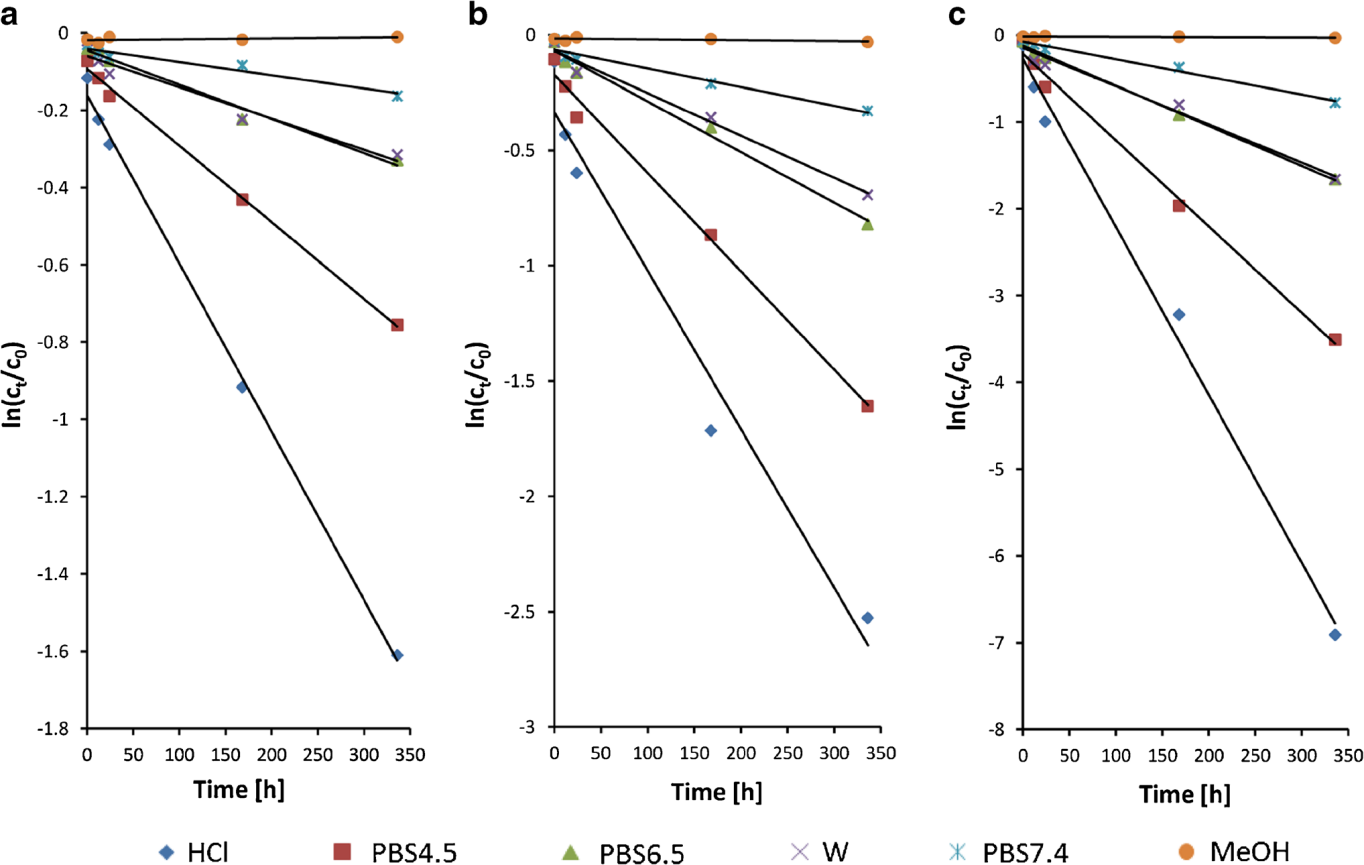
Pseudo-first-order plots showing the degradation of candesartan cilexetil at temperature: **a** 277 K, **b** 298 K, and **c** 310 K in aqueous solutions in the pH range 1.2–7.4 and in methanolic solution (MeOH); mean values are presented (*n* = 5; RSD < 10%)

CC in methanolic solution showed good stability with less than 3% of the total content loss at temperature of 310 K after 14 days (Fig. 3). In an earlier report, Ferreiros *et al.* [11] observed that CC degradation occurred in the methanolic solution only upon evaporation. The formation of candesartan and its ester was attributed to the basic hydrolysis of CC and transesterification reaction of the pro-drug. However, CC derivatives did not appear in our HPLC chromatograms when the methanolic solutions were heated but not evaporated to dryness.

#### Aqueous solutions—pH and temperature effect

The most important factors that affect the stability of substances in solutions are pH and temperature; therefore, the stability of CC at three different temperatures (277, 298, and 310 K ) and at five different pHs (1.2, 4.5, 6.5, 6.8, and 7.4) was investigated. Figure 3 presents the applicability of the pseudo-first-order model. The selected model showed a linear relationship between the logarithm of concentration *C*_*t*_/*C*_0_ and the storage time (*r* ^2^ in the range of 0.9979 to 0.8414 are presented in Table III). This indicates that the pseudo-first-order model can be used for predicting the kinetics of the CC degradation in aqueous solutions. The degradation rate profiles show fast decomposition in solution when temperature increases and pH decreases.

**Table III:** Rate Constants and Kinetic Parameters for Degradation of Candesartan Cilexetil in Aqueous Solutions Containing 0%, 0.35%, Or 0.7% of Polysorbate 20 (T20)

pH value		T20 0%			T20 0.35%			T20 0.7%		
	Temperature (K) parameter	277	298	310	277	298	310	277	298	310
1.0	*k*_obs_ (h^−1^)	4.36 × 10^−3>^	6.88 × 10^−3^	1.93 × 10^−2^	6.3 × 10^−4^	2.06 × 10^−3^	1.37 × 10^**−2**^	7.1 × 10^−4^	2.01 × 10^−3^	1.38 × 10^−2^
	*r* ^2^	0.9979	0.9699	0.9933	0.9532	0.9970	0.9935	0.9801	0.9861	0.9882
	*t*_0.5_ (h)	157.53	100.46	35.91	1,155.25	330.07	50.23	990.21	346.57	49.87
	*E*_a_ (kJ mol^−1^)	29.59			64.11			60.46		
4.5	*k*_obs_ (h^−1^)	1.98 × 10^−3^	4.27 × 10^−3^	9.93 × 10^−3^	1.3 × 10^−4^	3.5 × 10^−4^	4.77 × 10^−3^	1.4 × 10^−4^	5.3 × 10^−4^	4.77 × 10^−3^
	*r* ^2^	0.9971	0.9923	0.9936	0.8738	0.8820	0.9924	0.9177	0.9952	0.9953
	*t*_0.5_ (h)	346.57	161.20	70.01	6,931.47	1,732.87	144.41	6,931.47	1,386.29	135.91
	*E*_a_ (kJ mol^−1^)	33.27			78.43			80.57		
6.5	*k*_obs_ (h^−1^)	8.80 × 10^−4^	2.19 × 10^−3^	4.61 × 10^−3^	8.2 × 10^−5^	3.0 × 10^−4^	4.2 × 10^−3^	8.5 × 10^−5^	2.7 × 10^−4^	4.04 × 10^**−3**^
	*r* ^2^	0.9800	0.9871	0.9976	0.8615	0.8728	0.9918	0.9448	0.8414	0.9897
	*t*_0.5_ (h)	770.2	315.1	150.7	8,664.3	2,310.5	165.0	7,701.6	2,310.5	173.3
	*E*_a_ (kJ mol^−1^)	34.45			79.81			76.18		
W-6.8	*k*_obs_ (h^−1^)	8.0 × 10^−4^	1.84 × 10^−3^	4.39 × 10^−3^						
	*r* ^2^	0.9422	0.9845	0.9759						
	*t*_0.5_ (h)	866.43	385.08	1,57.53						
	*E*_a_ (kJ mol^−1^)	35.44								
7.4	*k*_obs_ (h^−1^)	3.40 × 10^−4^	8.1 × 10^−4^	2.03 × 10^−3^						
	*r* ^2^	0.9441	0.9477	0.9878						
	*t*_0.5_ (h)	2,310.49	866.43	346.57						
	*E*_a_ (kJ mol^−1^)	44.52								

*T20* polysorbate 20, *W-6.8* in water (pH 6.8), *k*_*obs*_ observed pseudo-first-order degradation rate constants, *r* ^*2*^ coefficient of determination in accordance with Eq. (1), *t*_*0.5*_ half-life (*t*_0.5_ = 0.693/*k*_obs_), *E*_*a*_ activation energy

In Table III, the calculated pseudo-first-order rate constants are listed showing the effect the temperature and pH have on the degradation rates. The pH-rate profiles reveal fast degradation of CC in acidic conditions. On the basis of the Arrehnius relationship (Eq. (2)), the linear plots of ln *k*_obs_ *versus* 1/T were used to calculate the energy of activation. The degradation rate of CC increases with a decreasing pH in the following order: PBS7.4 < W (pH 6.8) < PBS6.5 < PBS4.5 < HCl. The lowest energy of activation was observed for the acidic solution (pH 1.2) and it was 29.59 kJ/mol. The change of pH from 1.2 to 7.4 results in a 1.5-fold increase of activation energy and, depending on temperature, 8–14-fold decrease of degradation rate (*k*_obs_).

Change of temperature from 277 to 310 K caused 4.4 to 6.6-fold increase in the rate constants, depending on the solvent. After 24 h (310 K) in HCl (pH 1.2), the CC content was at 37.0% of the initial value while in PBS7.4 was at 83.5%. The half-lives of degradation were calculated to be from 1.5 day (pH 1.2, 310 K) to 96 days (pH 7.4, 277 K).

Accelerated conditions result in hydrolysis and formation of candesartan in an alkaline environment and desethyl CC in acidic and neutral conditions [12, 13, 18]. This is explained by the ionization occurring mainly above pH 6.3 (p*K*_*a*_ of CC). The resulting CC, in an ionized form, is highly susceptible to hydrolysis, which leads to the formation of candesartan, while in the nonionized form of CC, the ether bond between benzimidazole and ethyl moiety is broken [11, 12, 17].

**Fig. 4:**
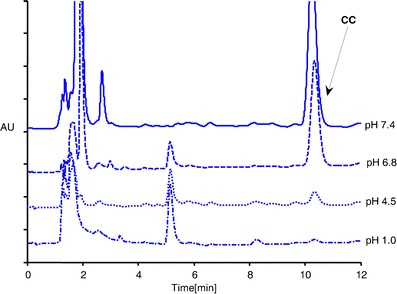
The HPLC chromatograms of candesartan cilexetil (*CC*) aqueous solutions (pH 1.2–7.4) after 14 days storage at 310 K

Figure 4 presents HPLC chromatograms of CC aqueous solutions after 14 days of storage at 310 K. According to literature data [11, 18], the peaks at retention times of 1.9 and 5.2 min were identified as a candesartan and desethyl CC, respectively. In acidic and neutral conditions (pH ≤6.8), an identical major degradation product was observed, represented by a peak with the retention time 5.2 min. Degradation of CC in PBS7.4 proceeded with the formation of a major degradation product, with a peak at the retention time of 1.9 min.

#### The effect of polysorbate 20

**Fig. 5:**
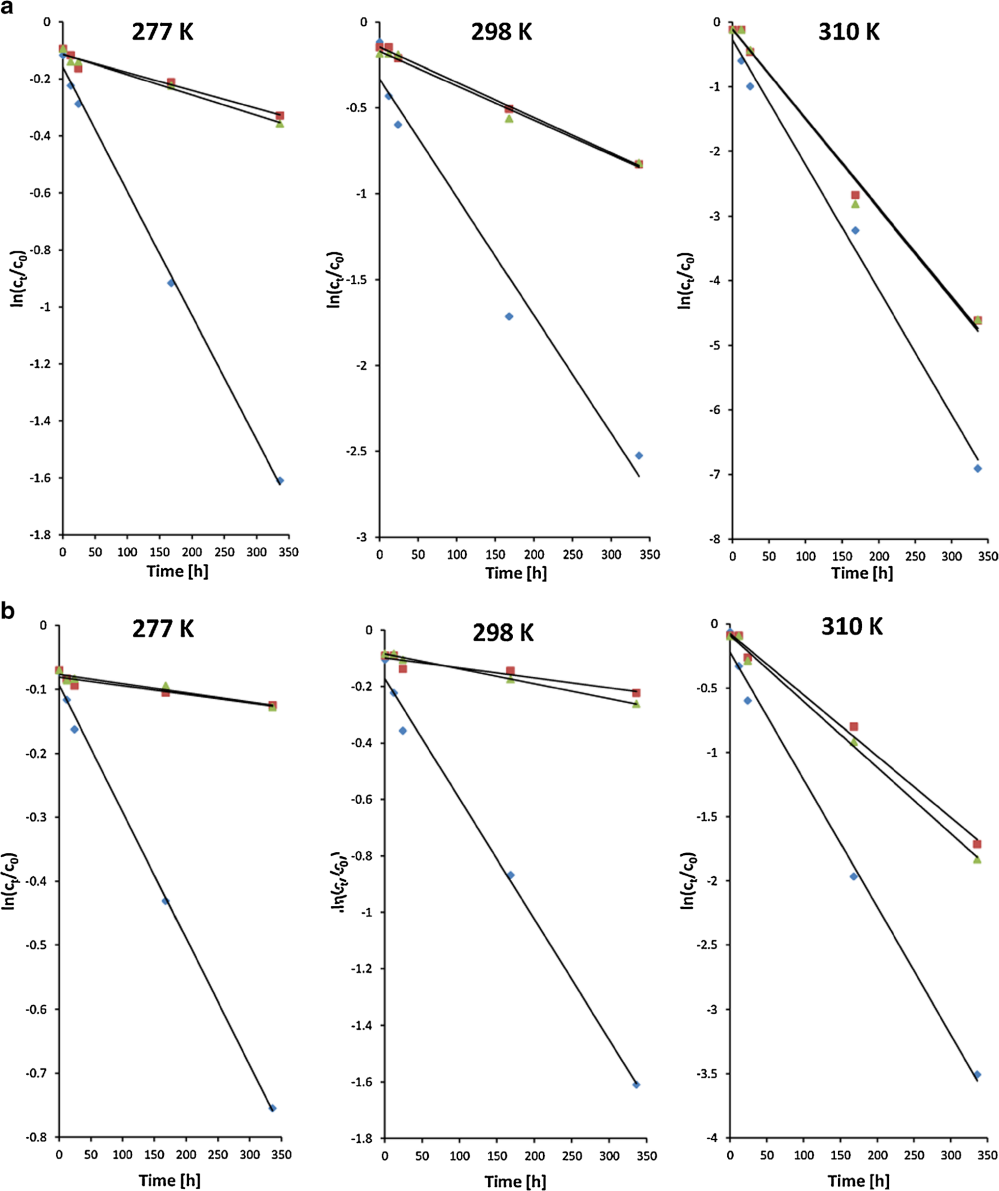

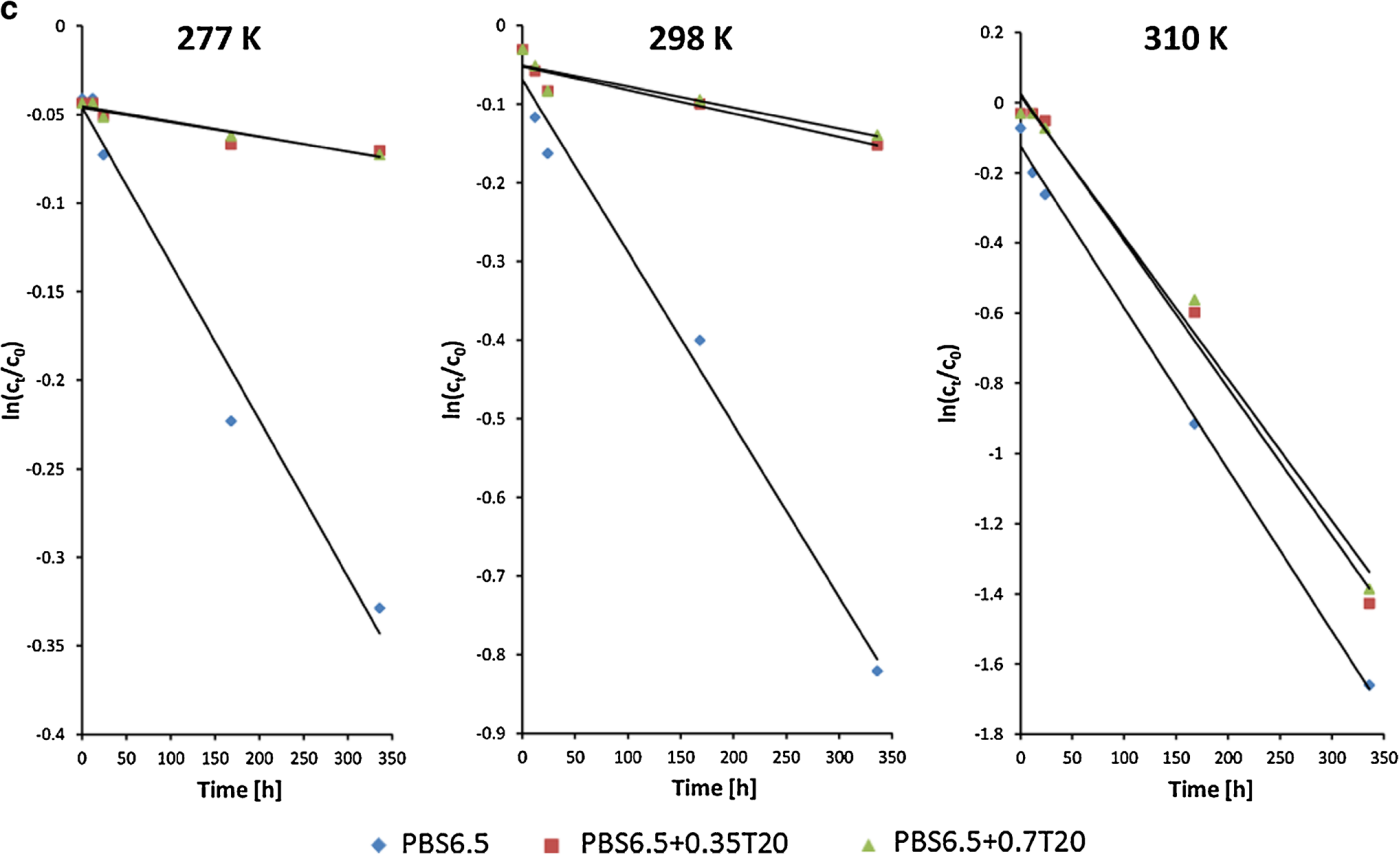
The effect of polysorbate 20 (T20 0.35 or 0.7% w/w) on degradation of candesartan cilexetil in HCl (**a**) and PBS buffers: pH 4.5 (**b**) and pH 6.5 (**c**) at temperature 277, 298, and 310 K; mean values are presented (*n* = 5; RSD < 10%)

Figure 5 and Table III demonstrate the effect of micellar solubilization of CC on its stability in an aqueous environment, dependent on pH and temperature. The protective effect of T20 is clearly visible, similar both in acidic and neutral solvents. The calculated energy of activation was approximately two times higher than for the solutions without T20, but the stability was not further increased with the increasing concentration of T20 from 0.35% to 0.7%.

The protective effect was decreasing, however, with an increased temperature and was only moderate or small at 310 K (relevant for a dissolution test). For example, in the presence of T20, in acidic conditions, *k*_obs_ decreased only by 28% and by 13% in pH 6.5.

Using the calculated *k*_obs_ values, one can estimate the degradation level occurring when a typical dissolution test is performed during 1 h (nonmodified drug release formulations) or during 24 h (modified dosage forms). If T20 is used (0.35% or 0.7%) in pH 6.5, one can expect the degradation to be at the level of 0.4% (1 h) and 9.3% (24 h). In acidic (HCl) conditions, these values are 1.4% and 28.3%, respectively. Thus, the dissolution test at pHs 1–6.8, required for a bio-waiver, may be performed for conventional tablets or capsules without the danger of degradation, but for longer tests, required for prolonged release formulations, the acceptor fluids at pH 6.5 or higher are suitable under the condition that the degradation is carefully monitored and the flow-through apparatus with a nondelayed analysis is recommended.

## CONCLUSIONS

CC is practically insoluble in water, and the addition of a surfactant is required to achieve the “sink” conditions during a dissolution test. T20, a surfactant recommended by FDA, dramatically increases the solubility of CC in a neutral phosphate buffer solution, while its effect at lower pH or in water is much smaller. The effect was not correlated, however, with the phosphate buffer concentration. The use of T20 0.35% solution in PBS at pH 6.5 enables to achieve the sink conditions in the volume of acceptor fluid even as small as 500 ml (for 32 mg CC doses), and this volume can be further reduced by using 0.7% of T20 solution in PBS. However, if a pH-dependent dissolution profile is necessary, the required volume of the acidic acceptor fluid is too large, since T20 does not increase CC solubility in acidic conditions so dramatically.

The choice of neutral pH for dissolution studies is also justified by the poor stability of CC in acidic conditions. T20 protects CC from degradation; this effect, however, is temperature-dependent and is practically nonrelevant at 310 K.

Due to a large solubilizing effect of T20 only in PBS solutions, despite of the insignificant effect of this surfactant on CC stability at 310 K, we were unable to propose a more appropriate acceptor fluid for CC dissolution studies than PBS at pH 6.5 with T20 at the concentration of up to 0.7%, which FDA recommends.
